# Nanopore Event-Transduction Signal Stabilization for Wide pH Range under Extreme Chaotrope Conditions

**DOI:** 10.3390/molecules21030346

**Published:** 2016-03-12

**Authors:** Stephen Winters-Hilt, Alexander Stoyanov

**Affiliations:** 1 Department of Biology, Connecticut College, Box #5564, 270 Mohegan Ave., New London, CT 06320, USA; 2 Department of Computer Science, Connecticut College, Box #5564, 270 Mohegan Ave., New London, CT 06320, USA; 3 Meta Logos Inc., 124 White Birch Dr., Guilford, CT 06437, USA; 4 Department of Pathology and Anatomical Sciences, College of Medicine, University of Missouri, 1 Hospital Dr., Columbia, MO 65202, USA; stoyanovA@health.missouri.edu

**Keywords:** channel current cheminformatics, nanopore detector, single-molecule biophysics, stationary signal analysis, biosensor

## Abstract

Operation of an α-hemolysin nanopore transduction detector is found to be surprisingly robust over a critical range of pH (6–9), including physiological pH = 7.4 and polymerase chain reaction (PCR) pH = 8.4, and extreme chaotrope concentration, including 5 M urea. The engineered transducer molecule that is captured in the standard α-hemolysin nanopore detector, to transform it into a transduction detector, appears to play a central role in this stabilization process by stabilizing the channel against gating during its capture. This enables the nanopore transduction detector to operate as a single molecule “nanoscope” in a wide range of conditions, where tracking on molecular state is possible in a variety of different environmental conditions. In the case of streptavidin biosensing, results are shown for detector operation when in the presence of extreme (5 M) urea concentration. Complications involving degenerate states are encountered at higher chaotrope concentrations, but since the degeneracy is only of order two, this is easily absorbed into the classification task as in prior work. This allows useful detector operation over a wide range of conditions relevant to biochemistry, biomedical engineering, and biotechnology.

## 1. Introduction

### 1.1. Channel Current Detectors

Early channel current detectors, known as Coulter Counters, had millimeter diameters (0.1 mm) and were used to count cell concentrations and mixture compositions [1]. Information obtained about the excluded cell volume was used in classifying blood cells as red or white, the ratio of which provided important data for medical diagnostics. The 100 μm pores of Coulter were devised in the early 1950s. It was not until the early 1970s that nanometer-scale pores were examined [2,3,4]. At that time, Bean made a nanometer-scale channel from crystalline structures (mica) that had defect tracks (from fission events). When etched with HF, the normally impervious mica is removed along the defect-track in its crystalline structure. Depending on how this process is controlled, pores have been obtained with diameters ranging down to 6 nm (50 nm diameter pores commercially available). Although this technology has been used for observations on uncharged particles (polystyrene spheres with 90 nm diameter, [3]), it does not work as well with charged molecules (like DNA). Another complication is that the etching method for pore construction inevitably leads to long tunnel-like channels, which does not provide the best configuration for detector uses. Detection of biomolecules with biologically-based nanometer-scale pores also showed promise at about this time with the work by Hladky and Haydon [5]. They showed that a biological channel, the bacterial antibiotic gramicidin, could self-assemble in a lipid bilayer to form a functional channel (with currents of order 1 pA). This potentially solved two of the mica-channel problems: the lipid bilayers are very thin, 1–10 nm across, and the protein-based, biologically functional, nanometer-scale pore seemed better suited to passing charged biomolecules. Gramicidin was too small to detect most biomolecules, however, since it could barely pass molecules the size of the water molecule. It was not until 1994 [6] that a sufficiently large pore was studied, α-hemolysin. In the 1994 paper, Bezrukov *et al.* studied the blockades of α-hemolysin resulting from a charge-neutral polymer: polyethylene glycol (PEG). Later modifications to the gramicidin pore permitted its use as an antibody-modulated (on-off) biosensor, while modifications to the α-hemolysin pore enabled its use as a metal biosensor, among other things.

Nanometer-scale pores are being developed in solid-state media [7,8] in hybrid media and with refinements to the biologically-based nanopore device. This provides rich opportunities for the future because at nanometer scale a wealth of new prospects arises, from assaying solutions, to recognizing individual molecular motions. Moving to solid state media, however, is a major undertaking since Nature, in the form of the α-hemolysin channel, has produced a very robust setting that is hard to match [7,8,9,10]. Simulation work is helping to clarify the problems in the solid-state setting [11], as well as offering insights into molecular interactions with the biological channels, including molecular ratchets [12].

In [13], nanopore-based sequencing is done on copy number variants in part of the human genome using the Oxford Nanopore Technologies MinION nanopore sequencer. Error rates for base-level detection on DNA constructs are explored in [14], and a review on nanopore translation-detection and hairpin-construct methods for DNA sequencing is given in [15]. DNA sequencing is a highly competitive field, however, where rival DNA sequencing technologies, such as at Illumina Inc., are well established, so translation-based nanopore sequencing may be very specialized in actual utility. Nonetheless, nanopore *biosensing*, including that based on short DNA-sequence detection (such as for miRNA detection), might be a critical area of strength of the different types of nanopore detector systems.

In the subsections that follow (Section 1.1.1, Section 1.1.2 and Section 1.1.3 and sections in Section 1.2), an introduction is given on a collection of sub-topics particularly relevant to the transduction detection approach, where the particular “transduction” mechanism indicated in this approach is further clarified by its various implementation and signal analysis advantages.

#### 1.1.1. Transduction *vs.* Translation

There are two ways to functionalize measurements of the flow (of something) through a “hole”: (1) translocation sensing; and (2) transduction sensing. The translocation methods in the literature are typically a form of a “Coulter Counter”, with a wide range of channel dimensions allowable, that typically measures molecules non-specifically via pulses in the current flow through a channel as each molecule translocates. The transduction biosensing method, on the other hand, requires nanopore sizes that are much more restricted, to the 1–10 nm inner diameters that might capture, and not translocate, most biomolecules. Transduction functionalization uses a channel flow modulator that also has a specific binding moiety, the transducer molecule. In transduction, the transducer molecule is used to measure molecular characteristics *indirectly*, by using a transducer/reporter molecule that binds to certain molecules, with subsequent distinctive blockade by the bound, or unbound, molecule complex. One such transducer, among many studied in [16,17,18,19,20,21,22], was a channel-captured dsDNA “gauge” that was covalently bound to an antibody. The transducer was designed to provide a blockade shift upon antigen binding to its exposed antibody binding sites. In turn, the dsDNA-antibody transducer platform then provides a means for directly observing the *single molecule* antigen-binding affinities of any antibody in single-molecule focused assays, in addition to detecting the presence of binding target in biosensing applications.

#### 1.1.2. Single-Molecule *vs.* Ensemble

When the extra-channel states correspond to bound or unbound, there are two protocols for how to set up the Nanopore Transduction Detection (NTD) platform: (1) observe a sampling of bound/unbound states, each sample only held for the length of time necessary for a high accuracy classification; or (2) hold and observe *a single* bound/unbound system and track its history of bound/unbound states. The single molecule binding history in (2) has significant utility in its own right, especially for observation of critical conformational change information not observable by any other methods (critical information for understanding antibodies, allosteric proteins, and many enzymes). The ensemble measurement approach in (1), however, is able to benefit from numerous further augmentations, and can be used with general transducer states, not just those that correspond to a bound/unbound extra-channel states.

Fundamentally, the weaknesses of the standard ensemble-based binding analysis methods are directly addressed with the single-molecule approach, even if only to do a more informed type of ensemble analysis. The role of conformational change during binding, in particular, could potentially be directly explored in this setting. This approach also offers advantages over other single-molecule translation-based nanopore detection approaches in that the transduction-based apparatus introduces two strong mechanisms for boosting sensitivity on single-molecule observation: (i) engineered enhancement to the device sensitivity via the transduction molecule itself; and (ii) machine learning based signal stabilization with highly sensitive state resolution. NTD used in conjunction with recently developed pattern recognition informed sampling capabilities [23] greatly extends the usage of the single-channel apparatus. For medicine and biology, NTD and machine learning methods may aid in understanding multi-component interactions (with co-factors), and aid in designing co-factors according to their ability to result in desired binding or modified state.

In ensemble *single-molecule* measurements (via serial detection process), the pattern recognition informed (PRI) sampling on molecular populations provides a means to accelerate the accumulation of kinetic information. PRI sampling over a population of molecules is also the basis for introducing a number of gain factors. In the ensemble detection with PRI approach [23], in particular, one can make use of antibody capture matrix and ELISA-like methods [16,17,18], to introduce two-state NTD modulators that have concentration-gain (in an antibody capture matrix) or concentration-with-enzyme-boost-gain (ELISA-like system, with production of NTD modulators by enzyme cleavage instead of activated fluorophore). In the latter systems the NTD modulator can have as “two-states”, cleaved and uncleaved binding moieties. UV- and enzyme-based cleavage methods on immobilized probe-target can be designed to produce a high-electrophoretic-contrast, non-immobilized, NTD modulator, that is strongly drawn to the channel to provide a “burst” NTD detection signal [16,17,18,22].

#### 1.1.3. Biomedicine Needs Biosensing with High Sensitivity in Presence of Interference

Clinical studies have shown an abundance of protein-based disease markers that accumulate in the blood of patients suffering from chronic kidney disease. In the case of the Bioscience PXRF01 marker, the stage of kidney disease is linearly correlated (r = 83) indicating that the more severe the disease, the greater the accumulation of the marker in the bloodstream of patients. The NTD biosensing platform provides a tool for quantifying the relationship between PXRF01 and its biosystem interactants with an unparalleled fidelity. With higher quantification of PXRF01 a more accurate characterization of the disease biomarker and kidney disease progression can be established. Greater sensitivity translates directly to earlier diagnosis and improved outcomes. The electrophoretic nature of the biosensing platform also allows for significant advantage in dealing with interference agents, whether in the blood sample itself, say, or due to contaminants, since the reporter molecule can be designed to have a charge that easily separates it from the interference agents (This is why blood can be scraped off the dirty floor at a crime scene and still accurately report on the identity or identities of those present.).

### 1.2. Nanopore Transduction Detection and Stochastic Carrier Wave Signal Analysis

The nanopore transduction molecule is engineered such that it can be individually captured in the channel with blockade signal consisting of a telegraph-like signal with stationary signal, and thus, statistics (often referred to as “stationary statistics”). This allows a system to be established where the longer the observation window the stronger the classification performance on the transducer molecule’s states, such as for bound and unbound states [24]. In a biosensing setting, NTD transducers can be introduced such that upon binding of analyte to the transducer molecule the toggling signal is greatly altered, to one with different transition timing and different blockade residence levels. The change in the channel blockade pattern, e.g., change in the modulatory signals statistics, is then identified using machine learning pattern recognition methods. The NTD approach has been shown to offer extraordinary sensitivity [16,17,18,19,20,21,22], but it is unclear how well it works in practice in the presence of interference agents (such as occur in blood) and with a wide range of buffers including the physiological pH range (for enzyme analysis) and buffers with high chaotrope concentration (for use in binding analysis). In this paper we demonstrate NTD signal stabilization is possible in each of these cases, with successful device operation obtained over a wide range of buffer pH and chaotrope concentration.

**Figure 1:**
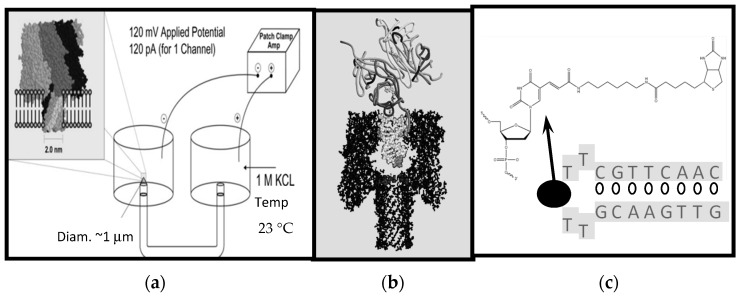
Schematic diagram of the Nanopore Transduction Detector [20]. (**a**) The nanopore detector consists of a single pore in a lipid bilayer which is created by the oligomerization of the staphylococcal alpha-hemolysin toxin in the left chamber, and a patch clamp amplifier capable of measuring pico Ampere channel currents located in the upper right-hand corner; (**b**) A biotinylated DNA hairpin molecule captured in the channel’s *cis*-vestibule, with streptavidin bound to the biotin linkage that is attached to the loop of the DNA hairpin; (**c**) The biotinylated DNA hairpin molecule (Bt-8gc) of (**b**).

In Figure 1a nanopore transduction detector is shown, in a configuration where the target analyte is streptavidin and biotin is used as the binding moiety (the fishing “lure”) at the transducer. In the absence of a transducer molecule and its target analyte, an open-channel current flows through the nanopore channel. When the appropriately charged transducer molecule is added, it is captured in the nanopore and disrupts the blockade current in a unique and measurable way as a result of its transient binding to the internal walls of the channel. In short, the transducer molecule “rattles” around inside the nanopore, imprinting its transient channel-binding kinetics on the channel blockade current and generating a unique signal: *A signal that is notably altered when the transducer is also bound to its target.*

#### 1.2.1. Ubiquitous Transduction Channel-Modulator Capability via Laser Modulation

**Figure 2:**
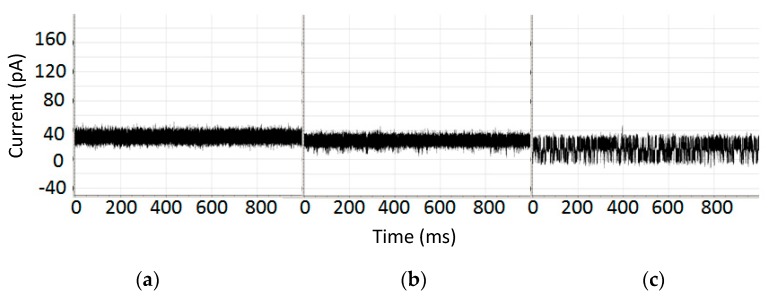
(**a**) Channel current blockade signal where the blockade is produced by 9GC DNA hairpin with 20 bp stem [19]; (**b**) Channel current blockade signal where the blockade is produced by 9GC 20 bp stem with magnetic bead attached; (**c**) Channel current blockade signal where the blockade is produced by c9GC 20 bp stem with magnetic bead attached and driven by a laser beam chopped at 4 Hz. Each graph shows the level of current in picoamps over time in milliseconds.

Biomolecules are in size-ranges that are well-sized for interaction with the α-hemolysin based nanopore detector shown in Figure 1. Duplex DNA cannot translocate the channel, for example, being captured at one end instead, but ssDNA can translocate. It is discussed in [25,26] that the end of the DNA molecule can be read for nine base-pair DNA molecules with very high accuracy based on the telegraph-like modulatory signals directly elicited during their channel interactions. DNA hairpins with lengths greater than roughly twelve base-pairs no longer elicit channel modulations, residing at a fixed-level blockade. If the high accuracy of the DNA terminus read can be extended to DNA hairpins at longer lengths, then highly efficient Sanger-style DNA sequencing might be possible on the Nanopore platform. In [16,17,18,19], a 20 base-pair hairpin with a magnetic bead attachment was studied with this in mind. The 20 base-pair hairpin (bphp) with magnetic bead produced a fixed level blockade that was similar to the blockade of the 20 bphp with no bead attachment (see Figure 2). In the presence of appropriate laser modulations with a chopped beam, channel blockade modulations resulted (Figure 2c). It was found that the modulatory signals were distinctive in this “re-awakened” configuration. Regarded in a different sense, the captured 20 bphp provides a terminus-dependent transform on the injected laser modulation that allows the terminus to be identified as in the 9 bphp analysis, presumably with similar high accuracy given sufficient observation time. Thus Sanger sequencing on the NTD platform appears possible with use of laser modulations (but without dyes). Perhaps what is more interesting, however, is simply that a molecule producing a fixed level blockade upon capture was successfully induced into a unique telegraph-like blockade signal by use of laser modulations.

Biomolecules in general, such as DNA, RNA, protein, and glycoprotein, typically provide channel blockades at a fixed level. If their blockades can be induced into telegraph-like signals via introduction of laser modulations, then the critical modulatory signal aspect of the transducer can be made ubiquitous, allowing close inspection of any molecule, via its states, when interacting with the nanopore.

#### 1.2.2. Antibody Binding Studies

Although some protein surface features clearly elicit blockade signals that are modulatory, not all surface features of interest will exhibit distinctive blockade signals when drawn to the channel and in these instances antibody or aptamer based targeting of those features could be used instead, where the antibody or aptamer is linked to a channel modulator that then reports on the presence of the targeted surface feature indirectly.

**Figure 3:**
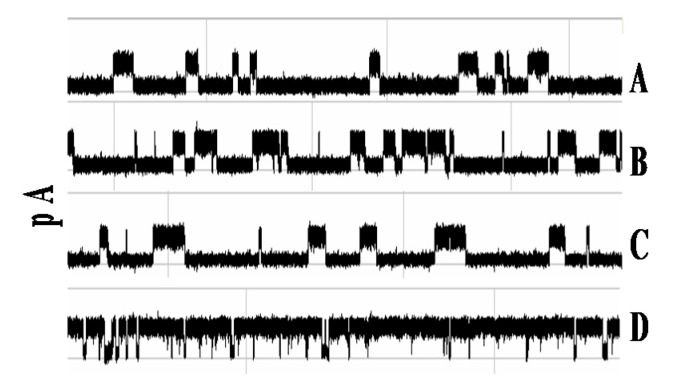
Antibody-Antigen binding—clear example from specific capture orientation [19]. Each trace shows the first 750 ms of a three-minute recording, beginning with the blockade signal by an antibody molecule that has inserted (some portion) into the α-hemolysin channel to produce a toggle signal (**A**–**C**). Antigen is introduced at the beginning of frame (**A**). Changes to the toggle signal are discernible in frame (**D**), indicating the binding event between the antibody and antigen has taken place.

In [19] it is found that the antibody blockade signal alters shortly after introduction of antigen, as Figure 3 shows upon addition of a moderately high concentration (100 µg/mL) of 200 kD multivalent synthetic polypeptide (Y,E)-A-K. Presumably, these changes are the result of antibody binding to antigen. The time before the blockade signal is altered is also interesting; it ranges from seconds to minutes (not shown). This presumably is a reflection of antibody affinity.

## 2. Background

The background subsections that follow describe some implementations of the NTD Nanoscope, and in each case it will be clear how the device utility is significantly enhanced if a larger operational range can be established in pH, chaotrope concentration, and interference concentration (as will then be shown Section 3). The NTD Nanoscope implementations discussed fall into four groups: (Section 2.1) annealing-based detection with use of chaotropes with application to: pathogen detection; miRNA detection and haplotyping; SNP detection and haplotyping; and Y-SNP based local sequencing; (Section 2.2) protein post-translational modification assaying, such as for performing glycoassays; (Section 2.3) enzyme studies; and (Section 2.4) high affinity/specificity (non-annealing) based biosensing, such as with aptamers and antibodies.

### 2.1. Pathogen Detection, miRNA Detection, and miRNA Haplotyping

In clinical diagnostics, as well as in biodefense testing, patient blood samples can be drawn for the purpose of assaying the DNA and glycoprotein contents. In the case of DNA there will be a preponderance of the individual’s own genomic DNA in such a sample, but if there is infection then trace amounts of the associated viral or bacterial DNA will be present as well. One of the questions that then arises is how to detect unique elements of bacterial DNA sequence with very high sensitivity and specificity. In [1] annealing-based detection is explored, where Y-shaped NTD transducer results are shown for tests involving an eight base ssDNA target [22]. The method can be extended to other lengths of targeted ssDNA, using annealing-based recognition. For longer lengths we can arrive at interesting detection scenarios for pathogens or for miRNAs (some possibly pathogenic). The known pathogen ssDNA targets could be longer, 15–25 bases say, to enable unique identifiers respective to a particular pathogen. For miRNA detection probes could be designed for ssDNA target annealing that is in the 7–15 base range.

MicroRNA detection follows a similar approach to the pathogen detection problem, but now typically working with a much shorter length nucleic acid detection target, a miRNA sequence based annealing target. In this setting, they often have similar “informed” analysis to pathogen detection analysis.

The detection of SNPs via annealing is demonstrated with the Y-shaped DNA transduction molecule that is minimally altered, and such that the SNP variant occurs in the Y-nexus region. In preliminary work with Y-transducers [19,22] we demonstrate how *single-base insertions or modifications at the nexus of the Y-shaped molecule can provide clearly discernible changes in channel-blockade signals*. The design of the Y-transducer for SNP detection was similar to the process mentioned in [19,22] for *na*nopore-detector *dir*ected (NADIR) searches for aptamers based on bound-state lifetime measurements. The NTD method provides a viable prospect for SNP variant detection to very high accuracy—possibly equaling the accuracy with which the NTD can discern DNA control hairpins that only differ in terminal base-pair (greater than 99.999% for sufficiently long observation time).

Y-DNA modulator platforms for biosensing can also provide a simple linker platform for use with antibody binding moieties, where a “linker” aptamer can be used that is covalently linked to the common base of the antibody (IgG) molecule (using a DNA tagged antibody approach). Aptamer tuning can also be enhanced in the nanopore setting using nanopore directed SELEX (referred to as NADIR in [19]), where binding strength can be selected to be not too strong or weak according to the desired tuning on the observed binding lifetimes, as seen in the state durations of the observed state noise.

Linkage of ssDNA to antibody is commonly done in immuno-PCR preparations, so another path with rapid deployment is to make use of a linkage technology that is already commoditized, e.g., a good NTD signal can then be produced with immuno-PCR tagged antibodies that are designed to anneal to another DNA molecule to form an NTD “Y-transducer”.

### 2.2. Glycoassayer, Posttranslational Assayer

Thyroid stimulating hormone (TSH) is present as a heterogeneous mixture of TSH molecules with different amounts of glycation (and other modifications). The extent of TSH glycation is a critical regulatory feedback mechanism. Tracking the heterogeneous populations of regulatory proteins is required to further our understanding and diagnostic capabilities for a vast number of diseases. Hemoglobin molecules are an example where specific, on-the-market, glycation diagnostics are in use—here extensive glycation is often associated with disease, where the A1c hemoglobin glycation test is typically what is performed in many over-the-counter blood monitors.

A nanopore-based glycoform assay could be performed on modified forms of the proteins of interest, *i.e.*, not just native, but deglycosylated, active-site “capped”, and other forms of the protein of interest, to enable a careful functional mapping of all surface modifications. Pursuant to this, the methodology could also be re-applied with digests of the protein of interest, to further isolate the locations of post-translational modifications when used in conjunction with other biochemistry methods.

Part of the complexity of glycoforms, and other modifications, of proteins such as hemoglobin and TSH, is that these glycoforms are present as a heterogeneous mixture, and it is the relative populations of the different glycoforms that may relate to clinical diagnosis or identification of disease. To this end, a protein’s heterogeneous mixture of glycations and other modified forms could be directly observed with the NTD Nanoscope setup, allowing direct access to the clinically relevant data of interest, not simply the concentration of one glycoform. Furthermore, it is the *transient*, dynamic, changes of the glycoform profile that is often the data of interest, such that a “real-time” profile of TSH glycoform populations are of clinical relevance, and obtaining such real-time profiling of modified forms (glycoforms, *etc.*) in physiological buffer conditions is an area of natural advantage for the NTD approach.

In conjunction with protein digests and HPLC, nanopore detection of glycation may provide a powerful new means to assay the post-translational modifications present for a given protein (in whole or via its digests), including their changing molecular complexations. This has profound significance for the understanding and treatment of a variety of diseases, including diabetes, where post-translational modifications to hemoglobin are an important biomarker for disease diagnosis and treatment.

### 2.3. Enzyme studies: HIV integrase

The NTD approach may provide an excellent method for examining enzymes, and other complex biomolecules, particularly their activity in the presence of different co-factors. There are two ways that these studies can be performed: (i) the enzyme is linked to the channel transducer, such that the enzyme’s binding and conformational change activity may be directly observed and tracked; or (ii) the enzyme’s substrate may be linked to the channel transducer and observation of enzyme activity on that substrate may then be examined. Case (i) provides a means to perform DNA sequencing if the enzyme is a nuclease, such as lambda exonuclease (discussed in Section 5.3). Case (ii) provides a means to do screening, for example, against HIV integrase activity (for drug discovery on HIV integrase inhibitors).

An example of a transient interaction that has been examined involves interaction of HIV integrase with its consensus DNA binding terminus [27]. One use of the nanoscope is as drug-discovery assayer in settings where measurements are made of transient interactions, such as HIV transcriptase interactions with DNA in the presence of interference agents or competitive inhibition molecules (decoy aptamers, for example).

HIV integrase binding to viral-DNA appears to favor the high flexibility of a CA/TG dinucleotide positioned precisely two base-pairs from the blunt terminus of the duplex viral DNA (and experimentally verified with the nanoscope in the conformational analysis shown in [28]). The CA/TG dinucleotide presence is a universal characteristic of retroviral genomes. Deletion of these base pairs impedes the integration process and it is believed that the unusual flexibility imparted by this base-pair on the terminus geometry is necessary for the binding to integrase. Once bound to integrase the viral DNA molecule is modified by removal of the two residues at the 3′-end together with subsequent insertion into the host genome.

### 2.4. Streptavidin Toxin Biosensor

The transducer molecule in the NTD “Streptavidin Toxin Biosensor” configuration (shown in Figure 1) consists of a bi-functional molecule: one end is captured in the nanopore channel while the other end is outside the channel. This exterior-channel end is engineered to bond to a specific target: the analyte being measured. When the outside portion is bound to the target, the molecular changes (conformational and charge) and environmental changes (current flow obstruction geometry and electro-osmotic flow) result in a change in the channel-binding kinetics of the portion that is captured in the channel. This change of kinetics generates a change in the channel blockade current which represents a signal unique to the target molecule.

**Figure 4:**
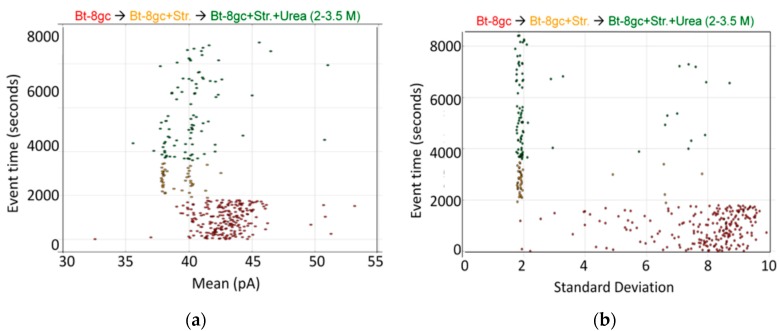
Biotinylated DNA hairpin transducer (Bt-8gc) in the presence of binding target (streptavidin) and chaotrope (urea). (**a**) Observations of individual blockade events are shown in terms of their blockade standard deviation (x-axis) and labeled by their observation time (y-axis) [20]. The standard deviation provides a good discriminatory parameter in this instance since the transducer molecules are engineered to have a notably higher standard deviation than typical noise or contaminant signals. At T = 0 s, 1.0 μM Bt-8gc is introduced and event tracking is shown on the horizontal axis via the individual blockade standard deviation values about their means. At T = 2000 s, 1.0 μM Streptavidin is introduced. Immediately thereafter, there is a shift in blockade signal classes observed to a quiescent blockade signal, as can be visually discerned. The new signal class is hypothesized to be due to (Streptavidin)-(Bt-8gc) bound-complex captures; (**b**) As with the Left Panel on the same data, a marked change in the Bt-8gc blockade observations is shown immediately upon introducing streptavidin at T = 2000 s, but with the mean feature we clearly see two distinctive and equally frequented (racemic) event categories. Introduction of chaotropic agents degrades first one, then both, of the event categories, as 2.0 M urea is introduced at T = 4000 s and steadily increased to 3.5 M urea at T = 8100 s.

Some of the transducer molecule results from [20] are shown in Figure 4, for a biotinylated DNA-hairpin that is engineered to generate two unique signals depending on whether or not a streptavidin molecule is bound.

In the NTD platform, sensitivity increases with observation time [24] in contrast to translocation technologies where the observation window is fixed to the time it takes for a molecule to move through the channel. Part of the sensitivity and versatility of the NTD platform derives from the ability to couple real-time adaptive signal processing algorithms to the complex blockade current signals generated by the captured transducer molecule. If used with the appropriately designed NTD transducers, NTD can provide excellent sensitivity and specificity and can be deployed in many applications where trace level detection is desired. The monoclonal antibody-based NTD system, deployed as a biosensor platform, possesses highly beneficial characteristics from multiple technologies: the specificity of monoclonal antibody binding, the sensitivity of an engineered channel modulator to specific environmental change, and the robustness of the electrophoresis platform in handling biological samples. In combination, the NTD platform can provide trace level detection for early diagnosis of disease as well as quantify the concentration of a target analyte or the presence and relative concentrations of multiple distinct analytes in a single sample.

In [20] a 0.17 µM streptavidin sensitivity is demonstrated in the presence of a 0.5 µM concentration of detection probes, with only a 100 s detection window. The detection probe is the biotinylated DNA-hairpin transducer molecule (Bt-8gc) described in Figure 1. In repeated experiments, the sensitivity limit ranges inversely to the concentration of detection probes (with PRI sampling) or the duration of detection window. The stock Bt-8gc has 1 mM concentration, so a 1.0 mM probe concentration is easily introduced (Note: The higher concentrations of transducer probes need not be expensive on the nanopore platform because the working volume can be very small: *cis* chamber volume is 70 µL, and could be reduced to 1.0 µL with use of microfluidics.). In [20] the selectivity of the detector in the presence of interference agents, such as albumin and sucrose and a variety of antibodies (without specific binding to biotin or the channel) was also examined, and a control transducer molecule with the same six-carbon linker arm from the DNA hairpin, but without the biotin “fishing lure” binding site, was introduced, where it was shown that no interaction (via change of blockade signal) was observed upon introduction of streptavidin, as expected.

## 3. Results

Results are shown for nanopore transduction detection based on biotin-streptavidin interactions (very strong), and antibody-antigen interactions. Extensive results are shown to validate the NTD Nanoscope results using standard methods from isoelectric focusing (IEF) gels and capillary electrophoresis (CE). Further results for the Streptavidin-Biotin biosensor, than those mentioned in Section 1.2 and Section 2.4, are shown in Section 3.1, confirming the NTD Nanoscope binding of Bt-8gc to streptavidin in urea with concentrations up to 5 M, where IEF Gel and CE validation results are found to be in agreement. In Section 3.2 new results on antibody binding are provided, along with validation results, building off the preliminary work mentioned in Section 1.2.2. The validation results show antibody binding with biotin as antigen in urea concentrations up to 2 M, with validation by IEF Gels.

### 3.1. Biotin-Streptavidin Binding Experiments

#### 3.1.1. The BT-8gc Transducer Retains its Viability in the Presence of Urea up to 5M Concentrations

**Figure 5:**
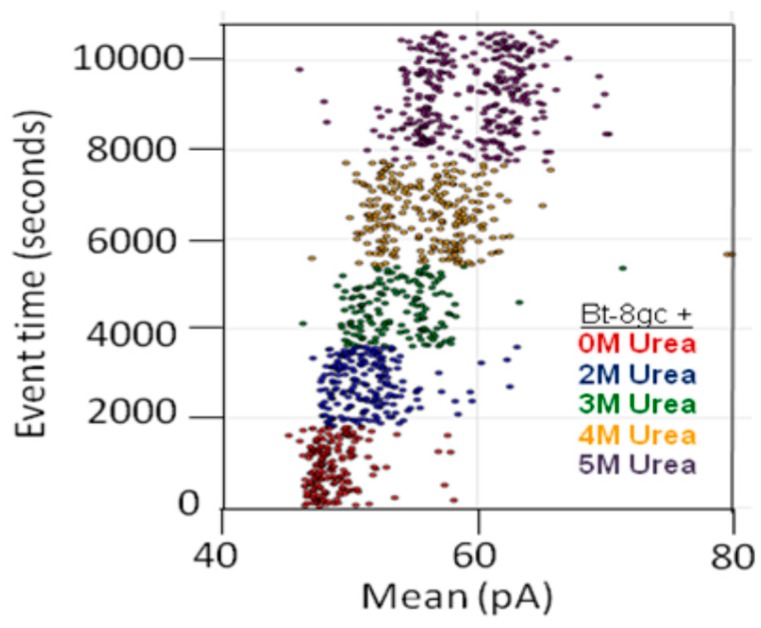
Transducer blockade event in increasing urea concentration. Sufficiently strong Urea concentration (5 M) results in racemization of the two loop capture-variants, while weaker urea (<2 M) does not. The results show Bt-8gc measurements at 30 min intervals (1800 s on vertical axis) with urea concentration 0, 2, and 3 M, 45 min at 4 M, and 60 min at 5 M, with signal blockade mean on the x-axis, with results consistent with the two-state loop hypothesis, and consistent with the observation of such in Figure 4 (see [20]) not due to zero or weak urea content but due to high strain due to mass and charge effects upon binding to the large streptavidin molecule.

In some instances, chaotropes (such as urea) are used to weaken the binding affinity, or DNA-DNA annealing affinity, of molecules studied with the nanoscope, such that binding tests can be performed with numerous on/off transitions in the lifetime of the experiment. In the case of DNA-DNA annealing, the collective binding that occurs can remain sufficiently strong in the presence of chaotrope such that it provides a clear contrast with non-collective binding interactions and can greatly improve signal quality. For this reason, and others, understanding the response of the channel and transducers in the presence of chaotropes is useful. The NTD approach will benefit most where the transducers provide little change, or have just a few states, when in channel blockade with change in chaotrope concentration. From high voltage capture strain prior studies it was found that the Bt-8gc blockades exhibit two different capture blockade signals. This is hypothesized to be due to two states of the transducer itself, probably due to two accessible loop “twists” conformations, one not normally accessible without capture-strain. Two transducer states that are degenerate (being simply due to hypothesized racemization on molecules with different loop conformations) is a manageable complication with the automated pattern recognition, but clearly reveals how at the single DNA-hairpin level of resolution we can see changes in molecular conformation (and terminus regions, as shown previously). Thus, a racemization over capture states with two loop “twists” was hypothesized to occur upon introduction of chaotropic agents (urea 2.0 M–5.0 M), and this result is confirmed in Figure 5 (A crude schematic for the twists is envisaged, from a top-down view of the hairpin loop, to look like the yin-yang symbol boundary that bows in to the left at the top, then to the right at the bottom, and the reverse for the other twist conformation.).

#### 3.1.2. Observations of Biotin-Streptavidin Binding on the NTD Nanoscope

Preliminary results on streptavidin biosensing were shown in Section 2.4 for urea concentration up to 3.5 M. The resolution of the bound/unbound Bt-8gc is greater than 99.99% accurate in less than 100 ms, with greater accuracy if longer observation time is used. The analysis uses the signal processing pipeline described in the Experimental Methods section on channel current cheminformatics where a 150-component feature extraction is done on each blockade signal. Using just two “human-friendly” features based on each signal’s maximum and minimum blockade values in that 100 ms observation window, a surprisingly clear separation of the molecular classes is easily discerned, as well as the role of urea in weakening interactions where bound states are reduced in observation frequency and unbound states increased. Initially, at 0 M urea, two clusters are easily discerned by eye. One corresponds to the Bt-8gc blockades, the other corresponds to the (Streptavidin)—(Bt-8gc) complex. Upon introduction of urea, signals for Bt-8gc unbound start to shift the Bt-8gc cluster, where direct quantification of the cluster results is directly accessible from the cheminformatics analysis.

#### 3.1.3. Bt-8gc—Streptavidin Binding Validation Using Gel Isoelectric Focusing (IEF) (3–10 pH Range) in Presence of Chaotropes

**Figure 6:**
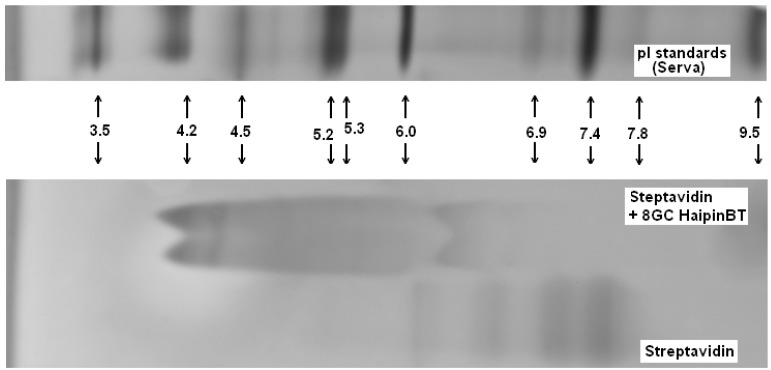
Biotinylated DNA hairpin (Bt-8gc) and streptavidin complex verification.

Complex formation between the biotinylated DNA hairpin (Bt-8gc) and streptavidin is shown on the NTD Nanoscope (Section 2.4 and Section 3.1.2)—this result is validated via isoelectric point shift analysis with isoelectric focusing in Figure 6. The standard gel analysis cannot resolve presence of different isoforms in a single “band” of gel, but Nanopore augmentation of gel electrophoretic methods, may offer a means to resolve components within the bands.

Electrophoretic methods provide a means to study the process of complex formation. Depending on the affinity (thermodynamic constant value) and the kinetics of the reaction, different electrophoretic techniques can be used. For highly stable complexes, the isoelectric focusing technique can be applied [29,30]. This electrophoresis technique has the advantage of extremely high resolution that allows maximally complete detection of existing heterogeneity in complex population, due to both multi-valent interaction and initial heterogeneity of interacting species.

**Figure 7:**
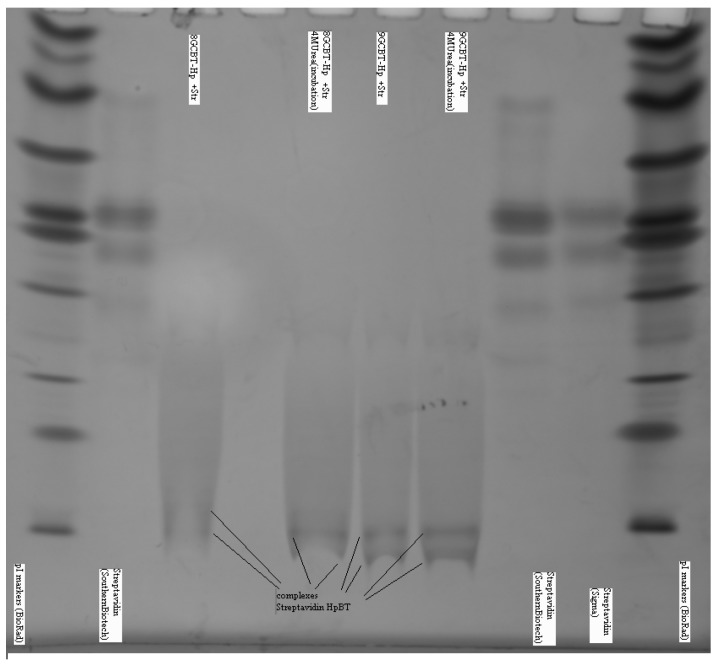
Complexes examined for streptavidin and biotinylated hairpins (Bt-8gc and Bt-9gc). Isoelectric focusing 3–10 pH range is implemented using a vertical system. Incubation time was 40min. Urea concentration in the sample buffer was 4 M (with no urea in the gel). Outer lanes: pI markers (BioRad). Inner lanes from left: (1) Streptavidin (Southern biotech); (2) Biotinylated 8GC hairpin + Streptavidin; (3) Biotinylated 8GC hairpin + Streptavidin + 4 M urea incubation; (4) Biotinylated 9GC hairpin + Streptavidin; (5) Biotinylated 9GC hairpin + Streptavidin + 4 M urea incubation; (6) Streptavidin (Southern biotech); and (7) Streptavidin (Sigma). The notation at the bottom of inner lanes (2–5) marks the complexes of streptavidin and the biotinylated DNA hairpins (The well pronounced pI—shift of the protein-hairpin complex is due to the presence of strong acidic moiety of DNA.).

In Figure 7 we show the gel IEF results describing the interaction of streptavidin and biotinylated hairpins. Due to very strong interaction between the streptavidin and biotin the complex is extremely stable: it does not break apart for hours and IEF detects practically no presence of free streptavidin. In Figure 7 the IEF spectra of streptavidin and streptavidin incubated with an excess of the biotinylated hairpins Bt-8gc and Bt-9gc, are shown. For the streptavidin, the two major components are visible (with their pIs at 7.1 and 7.5, approximately). After targeting with hairpin those streptavidin isoforms convert to two new bands (pI 4.2 and pI 4.35). We hypothesize that there exists a one to one correspondence between the two above pairs of major components (before and after the complexation takes place). According to our theoretical calculation such a high pI shift can be achieved when all four binding sites of the streptavidin molecule are targeted. Here we used the technique allowing for predicting the electric charge *vs.* pH relationship for a protein molecule based on the amino acid composition, or more generally, any biopolymer with known content of so-called ionogenic groups. The approach has limitations connected with the dissociation scheme selected for the model and the exact values of the dissociation, but typically serves as a reasonably good approximation for isoelectric point calculation or protein titration curve behavior [29,31]. The latter are often used as tool for optimizing various electrophoretic of chromatographic separations of intact or labeled proteins (with covalent or non-covalent interaction) [32,33]. With an excess of hapten, heterogeneity does not become more pronounced (although, by shorter incubation time or deficit or hairpin, some reaction products are detectable in the middle acidic range—pH 5–6.5).

One should expect that during the electrophoretic experiment, the reacting mixture becomes quickly divided to single components, so the complex is subjected to decay. The decay above, still, occurs rather slowly, as it can be seen in Figure 7. When the interaction is not as strong, the IEF method may not detect the complex formation. In particular, we did not detect any product that may correspond to a complex for anti-GFP Mab and its binding partner, GFP (data not shown).

#### 3.1.4. Bt-8gc—Streptavidin Binding Validation Using CE in the Presence of Chaotropes

We also used capillary electrophoresis (CE), as an alternative to IEF gel electrophoresis, since the CE processing time is much shorter, on the order of minutes. CE may be employed for analysis of fast chemical reactions (fast decay, *etc.*) [34,35,36]. Similar to chromatographic separation, capillary electrophoresis provides an opportunity to determine reaction kinetics [31,34,35,36] although the accuracy of these calculations is not very high. The CE technique also has certain advantages due to its suitability for study of complex formation at different pH and in presence of additives modulating the interaction (salt ions and other charged compounds). Results of CE experiments on (streptavidin)–(biotinylated hairpin) complexation have been obtained. The experiments aim to confirm complex formation, and its relative concentration decrease, under chaotropic conditions. Complex formation (streptavidin-Bt-8gc) is clearly exhibited as new peak appearance on electropherogram when the mixture of streptavidin and DNA is analyzed. It becomes possible to separate the same components, previously detected by gel IEF.

**Figure 8:**
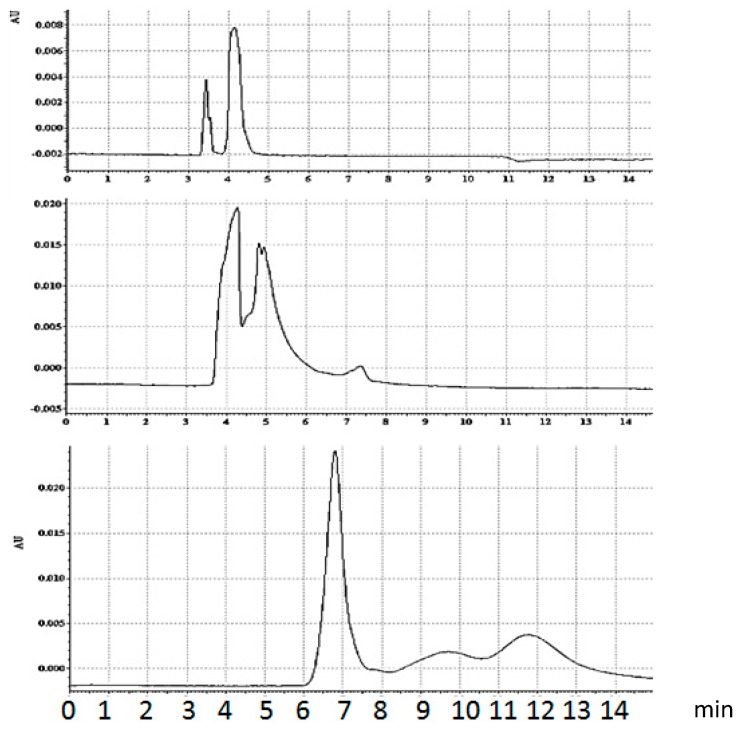
Capillary electrophoresis of equilibrium mixture, streptavidin/biotinylated hairpin (Bt-8gc) in presence of urea. Urea concentration increase suppresses the complex formation. Upper panel: 2.5 M urea running buffer. Sample—equilibrium mixture, no urea. Middle panel: 2.5 M urea running buffer. Sample—equilibrium mixture, 4 M urea. Left and right peaks on the two upper panels represent DNA and streptavidin-DNA complex, accordingly. The concentration of complex decreases with chaotrope concentration. In the case of 8 M urea concentration (lower panel) no complex formation is observed. The markings on the x-axes are in minutes.

The standard sample introduction scheme for two interactions substances is performed as a test: Streptavidin plug is introduced first (hydrodynamically), followed by the DNA plug. The two substances moving in the opposite directions interact very briefly, but sufficient to see side effects of complex formation. The complex has lower mobility and it is eluted second, after unbound DNA. The part of streptavidin which did not react with biotinylated DNA continues its moving towards anode and thus does not pass though the detector. In capillary electrophoresis of streptavidin/biotinylated hairpin (Bt-8gc) complex using sequential injection (Figure 8), a streptavidin sample plug is pressure introduced first, following by the second one of DNA. The DNA plug passes though the protein (streptavidin) and the interaction time is 2 s. By reducing the sample load and varying the DNA/protein ratio it was possible to separate two streptavidin-DNA complexes. (The more acidic complex, pI = 4.3 approximately, is eluted first). The existence of two major isoforms for complex is in accordance with our previous results on gel IEF. (Injection time/pressure from the bottom to the top: 5 s/0.5 psi (prot)–0.5 psa (DNA); 5 s/0.5 psi (prot)–0.3 psa (DNA); 5 s/0.2 psi (prot)–0.1 psa (DNA). Run at 250 Kv/cm.

By adding urea in the running buffer, even in the absence of urea in the sample buffer, one changes the electropherograms, beginning with indications of population shift, *i.e.*, different proportion between the complex and unbound hairpin. Further increase in urea concentration decreases the ratio between the complex and free Bt-8gc (here the concentration of urea in running buffer does not have a significant impact). Finally, very high urea concentration results in essential changes: the streptavidin is apparently mostly in its denatured form, although some capability of binding biotin still remains. Urea concentration increase influences the elution time. Several different effects act simultaneously, in particular, dielectric constant and viscosity change. In addition, there is a possibility of electroosmotic flow modulation. The most pronounced effect, apparently, is the conformation changes induced by urea; this explains considerable reduction in migration times both for denatured protein and DNA.

### 3.2. Biotin-mAb Binding Experiments

#### 3.2.1. Observations of Biotin-mAb Binding on the NTD Nanoscope

**Figure 9:**
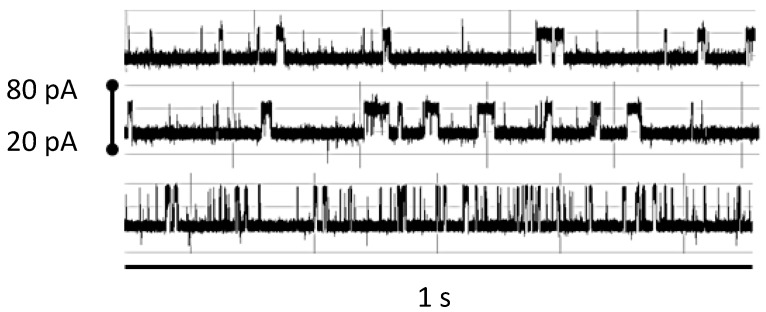
Robust NTD mAb-Bt-8gc (the mAb has biotin as antigen) binding signal under 100-fold biotin excess shows minimal interference effect (signals top middle show before after), while introduction of small amounts of chaotrope (<1 M urea) change the blockade signal significantly (bottom signal). Each signal is shown in a pA range from 20 to 80. The window of observation time is 1 s.

In one series of experiments, mentioned above, we used free antibody molecule interacting with the nanopore detector, where the antibody (anti-biotin) molecule is introduced to our nanopore device to produce the characteristic two-state telegraph signal (Figure 9). The blockade signal for the antigen is practically unaltered by excess antigen: even 100 fold excess of biotin does not change the blockade signal considerably (Figure 9). The signal changes greatly in presence of urea, however, in a relatively small concentration. Here the duration of any event to occupy upper state level becomes shorter and the total probability value of upper level decreases with urea concentration rise.

#### 3.2.2. Bt-8gc—mAb Binding Validation Using gel IEF (3–10) in presence of chaotropes

**Figure 10:**
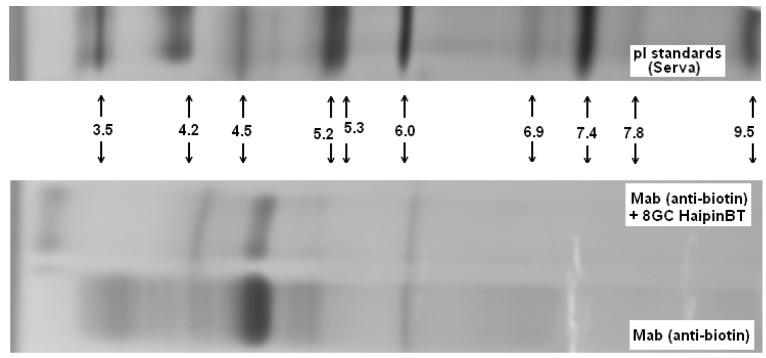
Biotinylated DNA hairpin (Bt-8gc) and mAb complex verification.

Complex formation between the biotinylated DNA hairpin (Bt-8gc) and mAb is shown on the NTD Nanoscope and validated via electrophoretic mobility shift analysis with isoelectric focusing in Figure 10.

**Figure 11:**
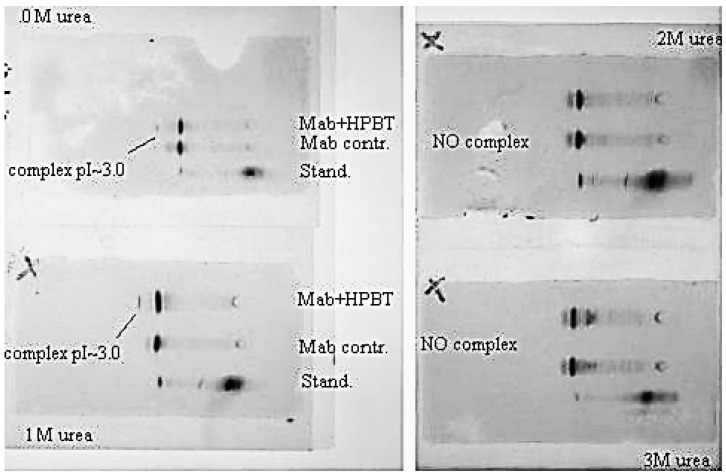
Urea suppresses complex formation between the Mab (anti-biotin) and biotinilated hairpin. Mab IEF spectra in presence of HP-BT (Bt-8gc in newer notation)) are shown at 0, 1, 2 and 3 M urea. The 1st and 2nd lines on each panel are Mab and Mab + HP-BT, correspondingly. Anode is on the left. Isoelectric focusing in 3–10 pH gradient (horizontal system).

With addition of chaotropic agents, Bt-8gc—mAb interactions are weakened, which results in a significant decrease in the relative concentration of the complex. We observe this in IEF experiments where the complexation in the system is seen between anti-biotin Mab and biotinylated hairpin in Figure 10. With progressive increase of urea the presence of complex becomes completely undetectable, as shown in Figure 11, where complex is no longer discerned at a urea concentration above 2 M.

### 3.3. Alpha-Hemolysin Nanoscope Operational pH Range

**Figure 12:**
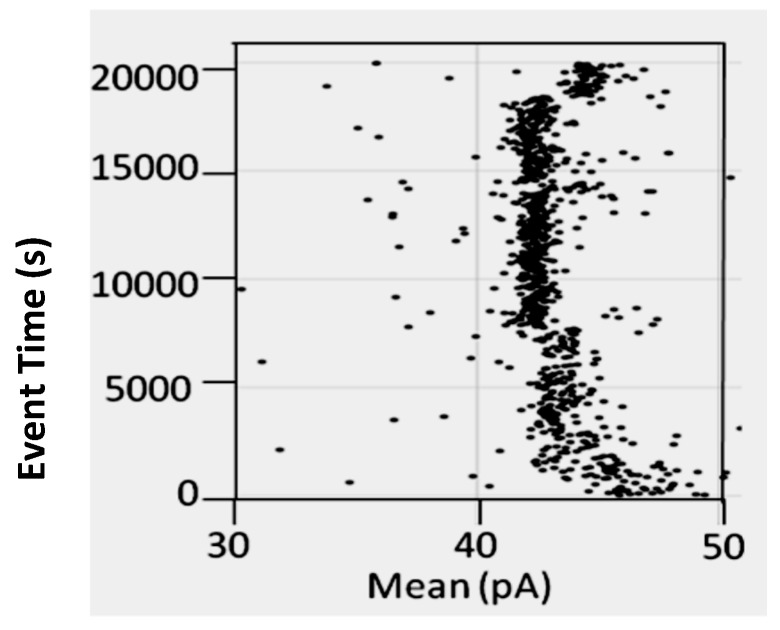
Part of the time-trace is shown for Bt-8gc events observed in an experiment that ran for two days. For T = 0 s to T = 3000 s the device is not at standard temperature and other conditions. At T = 3000 to 7500 s the channel operates in its normal, exposed chamber, evaporative mode, which leads to a concentration in the *cis*-chamber, including a concentration of salt (from 1.0 M KCl). At T = 3000 s, the standard detector is established, aside from having its operational pH set at 9.0 instead of 8.0. At T = 7500 s the channel changes configuration, and the hairpin signals obtained are now notably different in just the one attribute (mean). A brief return to a normal channel conductance occurs around T = 14,000 s, with a return to gated configuration at T = 14,500 s. A final return to normal channel conductance occurs around T = 18,000 s.

Since the nanopore detector we described was implemented using an alpha-hemolysin protein channel, the operational range of the detector is partly governed by the pH range over which the channel geometry remains relatively unchanged (see Figure 12). At pH 8.0 the channel is very stable with infrequent gating even when using higher voltages than 120 mV, or sampling frequencies above 10 Hz (with polarity switching on voltage). At pH 9.0 some gating does occur (a rare example is shown in Figure 12), but a surprisingly large range of pH appears to be accessible if the occurrences of channel gating can be ignored, or analyzed separately, or alleviated by introduction of the transducer molecule. Such is easily managed with the automation software, thereby allowing us to operate in a wide range of pH values, particularly those involving enzyme activity and protein-protein interactions. One beneficial characteristic is that channel gating and other complications appear to be further reduced when a transducer is captured. Evidently the captured, nearly channel-filling molecule, helps to stabilize the channel in its main conformation. In practice, minor partial gating in the channel under such conditions can be entirely absorbed into the pattern recognition task and be automatically handled with the pattern recognition pipeline.

Once the channel is established there exists the possibility of variation in composition of the upper electrode reservoir (according to the design we employed). Those changes allow for the possibility of regulating the protein-ligand interaction, as described earlier. It has to be mentioned that with changes in pH, viscosity, dielectric permeability *etc.*, one can influence not only the current trough the nanopore to some extent, but also the transport of the analyte of interest to the channel (or through the channel). While the letter effect mostly depends on electrophoretic phenomena, the effect of electroosmotic transport also has to be taken into account [37]. Additionally, during prolonged experiments, some effects of buffer electrolysis could potentially start playing an effect [37,38,39]. The latter may influence the local pH value inside the nanopore, especially when an experimental setup with different buffers in electrode chambers is considered.

## 4. Experimental Section

### 4.1. Nanopore Experiments

Each experiment is conducted using one α-hemolysin channel inserted into a diphytanoyl-phosphatidylcholine/hexadecane bilayer across a, typically, 20-micron-diameter horizontal Teflon aperture. The α-hemolysin pore has a 2.0 nm width allowing a dsDNA molecule to be captured while a ssDNA molecule translocates. The effective diameter of the bilayer ranges mainly between 5 and 25 μm (1 μm is the smallest examined). This value has some fluctuation depending on the condition of the aperture, which station is used (each nanopore station, there are four, has its own multiple aperture selections), and the bilayer applied on a day-to-day basis. Seventy-microliter chambers on either side of the bilayer contain 1.0 M KCl buffered at pH 8.0 (10 mM HEPES/KOH) except in the case of buffer experiments where the salt concentration, pH, or identity may be varied. Voltage is applied across the bilayer between Ag-AgCl electrodes. DNA control probes are added to the *cis* chamber at 10–20 nM final concentration. All experiments are maintained at room temperature (23 ± 0.1 °C), using a Peltier device.

### 4.2. Control Probe Design

Since the five DNA hairpins studied in the prototype experiment have been carefully characterized, they are used in the antibody (and other) experiments as highly sensitive controls. The nine base-pair hairpin molecules examined in the prototype experiment share an eight base-pair hairpin core sequence, with addition of one of the four permutations of Watson–Crick base-pairs that may exist at the blunt end terminus, *i.e.*, 5′-G|C-3′, 5′-C|G-3′, 5′-T|A-3', and 5′-A|T-3′. Denoted 9GC, 9CG, 9TA, and 9AT, respectively. The full sequence for the 9CG hairpin is 5′ CTTCGAACGTTTTCGTTCGAAG 3′, where the base-pairing region is underlined. The eight base-pair DNA hairpin is identical to the core nine base-pair subsequence, except the terminal base-pair is 5′-G|C-3′. The prediction that each hairpin would adopt one base-paired structure was tested and confirmed.

### 4.3. NTD-Aptamer Design

The Y-shaped NTD-aptamer molecule design we are currently using has a three-way DNA nexus geometry: 5’-CTCCGTCGAC GAGTTTATAGAC TTTT GTCTATAAACTC GCAGTCATGC TTTT GCATGACTGC GTCGACGGAG-3’. Two of the junctions’ arms terminate in a 4T-loop and the remaining arm, of length 10 base-pairs, is usually designed to be blunt ended (sometimes shorter with an overhang). The blunt ended arm has been designed such that when it is captured by the nanopore it produces a toggling blockade. One of the arms of the Y-shaped aptamer (Y-aptamer) has a TATA sequence, which is meant to be a binding target for TBP binding studies. In another, variant a DNA aptamer is placed at one arm, instead of a 4dT loop, and similarly with an HIV integrase consensus terminus sequence for use in studies of HIV integrase inhibitors. In general, any transcription factor binding site or DNA enzyme could be studied (or verified) in this manner.

### 4.4. Gel Electrophoresis and Image Analysis

Gel electrophoreis was performed mostly in vertical (Invitrogen, Eugene, OR, USA) or horizontal (Pharmacia, Uppsala, Sweden system. Alternatively, for IEF 11 cm IPG strips (Bio-Rad, Hercules, CA, USA) were used. The slab gels were fixed in ethanol/acetic acid mixture (10%/10%), stained with Comassie Blue or SyproRuby dye and further scanned at 100 dpi resolution using a Bio-Rad Molecular Imager FX. The resulting images were analyzed using the PDQuest software (Bio-Rad, V7.1).

### 4.5. Capillary Electrophoresis (CE)

CE was carried out with a P/ACE MDQ apparatus (Beckman Coulter, Brea, CA, USA) equipped with a UV detector. A 30 cm long, coated, low electro-osmotic flow, capillary with an inner diameter of 75 μm and outer diameter of 360 μm was used. The sample buffers and the electrophoresis run buffer were identical: 25 mM sodium tetraborate at pH. The capillary was rinsed with the run buffer for 5 min prior to each run. Electrophoresis was carried out for a total of 10 min by an electric field of 600 V/cm with a positive electrode at the injection end of the capillary. The temperature of the capillary was maintained at 10 ± 0.1 °C. At the end of each run, the capillary was rinsed with the same buffer at 10 psi for 2 min, followed by a rinse with deionized water for 5 min.

For sample injection, the inlet and outlet reservoirs are established with run buffer, and the capillary is prefilled with the run buffer. Normally, pressure injection 0.2–1.0 psi was used, or alternatively, sample injection was performed electro-kinetically.

### 4.6. Chemicals

Anti-biotin monoclonal antibodies obtained from Vector Laboratories (9100 (Hyb-8)) and from Stem Cell Technologies (#01405(C6D5.1.1), Burlingame, CA, USA) were used for binding studies. The antibodies, stored as supplied, were brought to a final dilution 1–4 μg/mL in the electrode chamber. Ampholytes (pH 4–9), and CE buffers were purchased from Bio-Rad. GFP was obtained from Molecular Probes (Eugene, OR, USA). Streptavidin was supplied by Sigma-Aldridge (St. Louis, MO, USA). Potassium chloride, HEPES and magnesium chloride were purchased from Sigma. Other chemicals were from Fisher Scientific, Atlanta, GA, USA.

### 4.7. Methods for Channel Current Cheminformatics

A combination HMM/EM-projection processing followed by time-domain FSA processing [22] allows for efficient extraction of kinetic feature information (e.g., the level duration distribution). One advantage of the HMM/EM processing is to reduce level fluctuations, while maintaining the position of the level transitions. The implementation uses HMM/EM parameterized with emission probabilities as Gaussians, which, for HMM/EM-projection, is biased with variance increased by approximately one standard deviations (see results shown). This method is referred to as HMM/EM projection because, to first order, it does a good job of reducing sub-structure noise while still maintaining the sub-structure transition timing. One benefit of this over purely time-domain FSA approaches is that the tuning parameters to extract the kinetic information are now much fewer and less sensitive (self-tuning possible in some cases).

The CCC processing is designed to rapidly extract useful information from noisy blockade signals using feature extraction protocols, Hidden Markov Models (HMMs) and Support Vector Machines (SVMs). For blockade signal acquisition and simple, time-domain, feature-extraction, a Finite State Automaton (FSA) approach is used that is based on tuning a variety of threshold parameters. The utility of a time-domain approach at the front-end of the signal analysis is that it permits precision control of the acquisition as well as extraction of fast time-scale signal characteristics.

Classification of feature vectors obtained by the HMM (for each individual blockade event) is then done using SVMs, an approach which automatically provides a decision hyperplane and a confidence parameter (the distance from that hyperplane) on each classification. SVMs are fast, easily trained, discriminators [22], for which strong discrimination is possible (without the over-fitting complications common to neural net discriminators). Different tools may be employed at each stage of the signal analysis in order to realize a robust (and noise resistant) tools for knowledge discovery, information extraction, and classification. Statistical methods for signal rejection using SVMs are also be employed in order to reject extremely noisy signals. Since the automated signal processing is based on a variety of machine-learning methods, it is highly adaptable to any type of channel blockade signal. This enables a new type of informatics (cheminformatics) based on channel current measurements, regardless of whether those measurements derive from biologically based or a semiconductor based channels.

Machine learning software has been integrated into the nanopore detector for “real-time” pattern-recognition informed (PRI) feedback [23]. The methods used to implement the PRI feedback include *distributed* HMM and SVM implementations, which enable the 100 fold to 1000 fold processing speedup that is needed.

## 5. Discussion

### 5.1. Validation of NTD Complexation Detection Using Standard Electrophoretic Techniques

We confirm the hypothesis that conventional electrophoretic methods (gel electrophoresis, IEF and SDS-electrophoresis), as well as capillary electrophoresis, can serve as excellent tools in guiding nanopore signal interpretation. With electrophoretic techniques, it has become possible to detect the complex formation, the number of different states (for multivalent systems) and, sometimes, the microheterogeneity of interacting molecules. Electrophoretic techniques are also an excellent tool for experimental monitoring the population distribution between different states as the concentration of chaotropic agent varies in the system. Since some traditional electrophoretic techniques require the presence of ionic detergent (SDS), however, they have limited application in studying the process of complex formation. With the validation results shown here we see how NTD methods, in turn, offer a means to inform and validate conventional electrophoretic methods, as well as offer an SDS free method for analyte separation according to molecular weight. In other words, nanopore detectors as specialty gels, where much of the gel representation of the information is recovered computationally, thus the name NTD *in-silico* gels.

### 5.2. Nanopore Detectors as In-Silico Gels

The idea here is that nanopore detectors may offer the separation/identification utility of gels, but under physiological buffer conditions and using non-destructive pattern recognition on blockade events to do the clustering “*in-silico*”. Using the PEG-shift approach described in [22], for example, the nanopore-based methods may be able to match the information content of drift-separation methods, such as mobility shift gels, but this will not resolve the topology mapping of the isoelectric focusing methods. Although a nanopore can be easily coupled to capillary electrophoresis geometries, for hybrid separation/clustering using capillary/nanopore, there is still no simple way for a *single* nanopore detector to “read” the focusing clusters without new plumbing being introduced, so will not be discussed further here.

### 5.3. Nanopore Detector Tolerance of Chaotropes and High Salt

The α-hemolysin channel demonstrates a high tolerance to high salt concentration and the presence of chaotropic agents, which is important to establish a platform for the study of binding between other molecules under such conditions. By varying the composition of running buffer it is possible to control the interaction of analyzed molecules with the nanopore or with each other. We performed tests of the impact on binding affinity between streptavidin, or mAb, and biotin upon introduction of chaotropic agents. This provides new opportunities in nanopore detector applications.

### 5.4. Purity Tests

The protein species examined were subjected to a careful purity tests in order to determine the presence of contamination and existence of microheterogeneity (if any). The controls included electrophoretic techniques: IEF and SDS electrophoresis in gel and microchip (Agilent). The IEF analysis shows subtle differences in isoelectric point values, while no heterogeneity is revealed by SDS electrophoresis. We observed microheterogeneity for the monoclonal antibodies (Mabs) we analyzed. The different mAb’s exhibit non-similar IEF-spectra and different levels of contamination. In addition, they differ by the degree of glycosylation (MB-9100 show much higher sugar content being stained Pro-Q-Emerald stain). This difference possibly explains particular features of IEF spectra for these Mabs and the IEF spectra changes in presence of urea.

To discriminate the contribution coming from the low MW impurities we tested a number of such substances: amphoteric dyes, polypeptides and neutral compounds (PEG), in order to recognize such contribution in the future when we are dealing with the signal processing. The electrophoretic mobility tests are consistent with the binding results observed using the NTD method.

We successfully employed electrokinetic method for controlling the purity heterogeneity and complex formation analysis. We anticipate further progress can be connected with future use of mass-spectroscopy [40,41].

### 5.5. NTD Capabilities and Limitations

The NTD idea is a noise-state transduction detection method, and can even be used in very-low-current nanopore transduction detection (NTD), where laser pulsing can be used to induce the coherency modulation, if not already present, in the observed channel current noise that is monitored for transducer state change. This is a generalized channel transduction detection setting insofar as the stationary statistical profiles are obtained from the stationary noise fluctuations induced via laser modulation of the channel’s environment (not the channel’s DC ionic current observations). In the general device-enhancing setting, any introduction of system modulations that results in stationary signals with stationary statistical profiles can be leveraged in a similar manner. One application of note is to live whole-cell studies, where large fertilized sea urchin egg cells, for example, provide a very accessible and well-studied model biological system for complex biosystem analysis, where a single sea urchin egg cell could be merged directly onto the operational NTD bilayer/aperture (with transducer in place), and non-destructive live-cell cytosol assaying might be possible.

The NTD idea also relates to single-molecule analysis and characterization using the nanopore transduction detection method and the stochastic carrier wave signal analysis method, whereby real-time assaying of transient molecular complexes, such as glycoprotein complexes, and intricate protein-protein interactions, such as STAT dimerization, can be done. Single-molecule based analysis, performed sequentially on captured analytes, may allow NTD-glycoassays to be performed on blood samples for a single test to provide detailed analysis of the individual’s globin glycosylations (Section 5.2). The NTD setting can also be used in gene-circuit analysis using a biosystem extra element theorem (EET) analysis method together with a method for non-destructive analysis of gene interaction networks using NTD with a weakly binding reporter molecule.

By use of pre-processing with simple capture matrices, or microarrays co-opted for that purpose (if nucleic acid involved), it also appears possible to perform detection on very low concentration analytes, with application in broad-based pathogen exposure assays, miRNA detection and haplotyping schemes, and SNP detection and haplotyping schemes, that were mentioned in the Background, and when capture matrices do not suffice, such as with membrane bound analytes of interest, the TERISA and TARISA methods can be used in pre-processing instead [22].

### 5.6. Processive DNA Enzyme with Laser/Dye info for Enhanced Fidelity for Very Long Reads

Lambda-exonuclease, with sufficient magnesium and other buffer conditions, will processively remove nucleotides from the 3-prime strand of a dsDNA molecule. In one implementation of the nanoscope for purposes of DNA sequencing, a nanopore transduction molecule can be introduced that consists of a DNA hairpin channel modulator linked (covalently) to a lambda exonuclease molecule. Upon introduction of a substrate of dsDNA and buffer conditions suitable for lambda-nuclease interactions (buffer conditions where both nanopore channel and lambda-exonuclease are functional are already known to exist), it is possible to measure the changes in channel blockade modulations that occur during the exonuclease activity.

If working with a lambda-exonuclease molecule directly above the channel opening, with a linkage to a captured channel modulator, by introducing duplex DNA substrate with appropriate co-factors (magnesium, *etc.*) it may be possible to enable the enzymatic activity of the lambda-exonuclease based channel modulator. With this arrangement (discussed further in [22]) we have an added coincidence detection arrangement that may enable DNA sequencing to be directly performed at the single molecule level: detection event 1 is via the transduction modulation accompanying the enzymatic clipping activity on a particular base (or modified base); detection event 2 will be the modulation of the channel current resulting from the passage of the clipped DNA base past the channel-with DNA-hairpin configuration (preliminary tests, not published, with DNA hairpins and individual bases show that these translocations can and do occur, are clearly observable, but not strongly distinguishable by themselves, at least with the buffer conditions examined).

## 6. Conclusions

The engineered transducer molecule central to the transduction approach is shown to offer the added benefit of channel stabilization, and thus overall device stabilization, when working with buffer conditions involving extreme pH, chaotrope, or interference concentration. This enables the nanopore transduction detector to operate as a single molecule “nanoscope” in a wide range of conditions, where what is seen is not the molecule in a visual sense as with the microscope, but molecular state, where tracking on molecular state is critical to a complete understanding of many allosteric proteins and enzymes. This allows useful device operation in a wide range of conditions relevant to biochemistry, biomedical engineering, and biotechnology.

Binding affinity results upon introduction of chaotropic agents (2.0–3.5 M urea) show agreement between nanopore transduction detection (NTD) and standard electrophoretic-separation methods, including: (i) isoelectric focusing; and (ii) capillary zone electrophoresis.

## References

[1] Coulter W.H. (1953). 1953. Means for Counting Particles Suspended in a Fluid. U.S. Patent.

[2] DeBlois R.W., Bean C.P. (1970). Counting and sizing of submicron particles by the resistive pulse technique. Rev. Sci. Instrum..

[3] Bean C.P., Eisenman G. (1972). The physics of porous membranes—Neutral pores. Membranes.

[4] DeBlois R.W., Bean C.P., Wesley R.K.A. (1977). Electrokinetic measurements with submicron particles and pores by the resistive pulse technique. J. Colloid Interface Sci..

[5] Hladky S.B., Haydon D.A. (1972). Ion transfer across lipid membranes in the presence of gramicidin A. Biochim. Biophys. Acta.

[6] Bezrukov S.M., Vodyanoy I., Parsegian V.A. (1994). Counting polymers moving through a single ion channel. Nature.

[7] Li J., McMullan C., Stein D., Branton D., Golovchenko J. (2001). Solid state nanopores for single molecule detection. Biophys. J..

[8] Li J., Stein D., McMullan C., Branton D., Aziz M.J., Golovchenko J.A. (2001). Ion beam sculpting at nanometer length scales. Nature.

[9] Song L., Hobaugh M.R., Shustak C., Cheley S., Bayley H., Gouaux J.E. (1996). Structure of staphylococcal α-hemolysin, a heptameric transmembrane pore. Science.

[10] Kullman L., Winterhalter M., Bezrukov S.M. (2002). Transport of maltodextrins through maltoporin: A single-channel study. Biophys. J..

[11] Palonsky S., Rossnagel S., Stolovitsky G. (2007). Nanopore in metal-dielectric sandwich for DNA positional control. Appl. Phys. Lett..

[12] Luan B., Pemg H., Polonsky S., Rossnagel S., Stolovitsky G., Martyna G. (2010). Base-by-Base ratcheting of single stranded DNA through a solid-state nanopore. Phys. Rev. Lett..

[13] Jain M., Fiddes I.T., Miga K.H., Olsen H., Paten B., Akeson M. (2015). Improved data analysis for the MinION nanoipore sequencer. Nat. Methods.

[14] Schreiber J., Wescoe Z.L., Abu-Shumays R., Vivian J.T., Baatar B., Karplus K., Akeson M. (2013). Error rates for nanopore discrimination among cytosine, methylcytosine, and hydroxymethylcytosine along individual DNA strands. Proc. Natl. Acad. Sci. USA.

[15] Ying Y.-L., Zhang J., Gao R., Long Y.-T. (2013). Nanopore-based sequencing and detection of nucleic acids. Angew. Chem. Int. Ed..

[16] Winters-Hilt S., Pincus S. (2012). Nanopore-Based Biosensing.

[17] Winters-Hilt S., and Pincus S. (2007). Channel Current Cheminformatics and Bioengineering Methods for Immunological Screening, Single-Molecule Analysis, and Single Molecular-Interaction Analysis.

[18] Winters-Hilt S. (2015). Channel Current Cheminformatics and Bioengineering Methods for Immunological Screening, Single-Molecule Analysis, and Single Molecular-Interaction Analysis.

[19] Winters-Hilt S. (2006). Nanopore Detector based analysis of single-molecule conformational kinetics and binding interactions. BMC Bioinform..

[20] Winters-Hilt S., Horton-Chao E., Morales E. (2011). The NTD Nanoscope: potential applications and implementations. BMC Bioinform..

[21] Winters-Hilt S., Adelman R. (2013). Method and System for Characterizing or Identifying Molecules and Molecular Mixtures. U.S. Patent.

[22] Winters-Hilt S. (2011). Machine-Learning Based Sequence Analysis, Bioinformatics & Nanopore Transduction Detectio.

[23] Eren A.M., Amin I., Alba A., Morales E., Stoyanov A., Winters-Hilt S. (2010). Pattern recognition informed feedback for nanopore detector cheminformatics. Adv. Exp. Med. Biol..

[24] Winters-Hilt S. Method and System for Stochastic Carrier Wave Communications, Radio-Noise Embedded Steganography, and Robust Self-Tuning Signal Discovery and Data-Mining. US Patent.

[25] Vercoutere W., Winters-Hilt S., Olsen H., Deamer D., Haussler D., Akeson M. (2001). Rapid discrimination among individual DNA molecules at single nucleotide resolution using an ion channel. Nat. Biotechnol..

[26] Winters-Hilt S., Vercoutere W., DeGuzman V.S., Deamer D., Akeson M., Haussler D. (2003). Highly accurate classification of watson-crick base-pairs on termini of single DNA molecules. Biophys. J..

[27] Winters-Hilt S., Davis A., Amin I., Morales E. (2007). Nanopore current transduction analysis of protein binding to non-terminal and terminal DNA regions: Analysis of transcription factor binding, retroviral DNA terminus dynamics, and retroviral integrase-DNA binding. BMC Bioinform..

[28] Winters-Hilt S., Landry M., Akeson M., Tanase M., Amin I., Coombs A., Morales E., Millet J., Baribault C., Sendamangalam S. (2006). Cheminformatics methods for novel nanopore analysis of HIV DNA termini. BMC Bioinform..

[29] Righetti P.G., Stoyanov A.V., Zhukov M. (2001). The Proteome Revisited.

[30] Cann J.R., Stimpson D.I., Cox D.J. (1978). Isoelectric focusing of interacting systems: Carrier ampholyte-induced macromolecular association or dissociation into subunits. Anal. Biochem..

[31] Kabytaev K., Durairaj A., Shin D., Rohlfing C.L., Connolly S., Little R.R., Stoyanov A.V. (2016). Two-step ion-exchange chromatographic purification combined with reversed-phase chromatography to isolate C-peptide for mass spectrometric analysis. J. Sep. Sci..

[32] Stoyanov A.V., Rohlfing C.L., Connolly S., Roberts M.L., Nauser C.L., Little R.R. (2011). Use of cation exchange chromatography for human C-peptide isotope dilution–mass spectrometric assay. J. Chromatogr. A.

[33] Stoyanov A.V., Righetti P.G. (1987). Ampholyte dissociation theory and properties of ampholyte aqueous solutions. Electrophoresis.

[34] Andreev V.P., Makarova E., Pliss N.S. (2001). New capability of electroinjection analysis: Investigation of chemical reaction kinetics. Anal. Chem..

[35] Patterson D.H., Harmon B.J., Regnier F.E. (1996). Dynamic modeling of electrophoretically mediated microanalysis. J. Chromatogr. A.

[36] Shalongo W., Jagannadham M., Stellwagen E. (1993). Kinetic analysis of the hydrodynamic transition accompanying protein folding using size exclusion chromatography. Comparison of spectral and chromatographic kinetic analyses. Biopolymers.

[37] Stoyanov A.V., Righetti P.G. (1999). Steady-state electrolysis of a solution of nonamphotheric compounds. Electrophoresis.

[38] Stoyanov A.V., Kuai S., Meng J. (2010). Water electrolysis in mono-and hetero-phase low conductive systems and a secondary pH gradient establishment. Electrolysis: Theory, Types and Applications.

[39] Stoyanov A.V., Pawliszyn J. (2004). Buffer composition changes in background electrolyte during electrophoretic run in capillary zone electrophoresis. Analyst.

[40] Stoyanov A. (2012). IEF-based multidimensional applications in proteomics: Toward higher resolution. Electrophoresis.

[41] Stoyanov A.V., Rogatsky E., Stein D., Connolly S., Rohlfing C.L., Little R.R. (2013). Isotope dilution assay in peptide quantification: The challenge of microheterogeneity of internal standard. Proteom. Clin. Appl..

